# Interaction of Type IV Toxin/Antitoxin Systems in Cryptic Prophages of *Escherichia coli* K-12

**DOI:** 10.3390/toxins9030077

**Published:** 2017-03-01

**Authors:** Zhongling Wen, Pengxia Wang, Chenglong Sun, Yunxue Guo, Xiaoxue Wang

**Affiliations:** 1 Key Laboratory of Tropical Marine Bio-resources and Ecology, Guangdong Key Laboratory of Marine Materia Medica, Research Network for Applied Microbiology (RNAM) Center for Marine Microbiology, South China Sea Institute of Oceanology, Chinese Academy of Sciences, Guangzhou 510301, China; zlwen@scsio.ac.cn (Z.W.); wangpengxia@scsio.ac.cn (P.W.); clsun@scsio.ac.cn (C.S.); yunxueguo@scsio.ac.cn (Y.G.); 2 University of Chinese Academy of Sciences, Beijing 100049, China

**Keywords:** type IV toxin/antitoxin, prophage, *Escherichia coli*

## Abstract

Toxin/antitoxin (TA) systems are widespread in prokaryotic chromosomes and in mobile genetic elements including plasmids and prophages. The first characterized Type IV TA system CbtA/CbeA was found in cryptic prophage CP4-44 in *Escherichia coli* K-12. Two homologous TA loci of CbtA/CbeA also reside in cryptic prophages of *E. coli* K-12, YkfI/YafW in CP4-6 and YpjF/YfjZ in CP4-57. In this study, we demonstrated that YkfI and YpjF inhibited cell growth and led to the formation of “lemon-shaped” cells. Prolonged overproduction of YkfI led to the formation of “gourd-shaped” cells and immediate cell lysis. YafW and YfjZ can neutralize the toxicity of YkfI or YpjF. Furthermore, we found that YkfI and YpjF interacted with cell division protein FtsZ in *E. coli*, but ectopic expression in *Pseudomonas* and *Shewanella* did not cause the formation of “lemon-shaped” cells. Moreover, deletion of all of the three toxin genes together decreased resistance to oxidative stress and deletion of the antitoxin genes increased early biofilm formation. Collectively, these results demonstrated that the homologous Type IV TA systems in *E. coli* may target cell division protein FtsZ in *E. coli* and may have different physiological functions in *E. coli*.

## 1. Introduction

The first toxin/antitoxin (TA) system, CcdA/CcdB, was described in 1983 as a module enhancing the stability of the F plasmid by post-segregational killing of plasmid-free daughter cells [1]. Subsequently, other plasmid-encoded TA systems were identified by their ability to enhance plasmid stability in addition to a number of TA systems detected by homology searches in bacterial chromosomes. Intriguingly, bacteria were identified that contain dozens of putative TA systems per genome; for instance, more than thirty TA systems have been characterized in the commensal *Escherichia coli* K-12 strain [2], and more than seventy TAs have been identified in the human pathogen *Mycobacterium tuberculosis* [3]. TA systems participate in many important cellular processes including transcription and translation, thus affecting cell physiology including biofilm formation, phage inhibition, persistence, and stress responses [4,5,6,7,8]. Several studies have shown that TA systems promote the maintenance of the mobile genetic elements such as integrative conjugative elements in the bacterial hosts [9,10]. Based on the nature and mode of action of antitoxins, TA systems have been classified into six different types [11]. The product of the toxin gene is a protein, while the product of the antitoxin is either a protein (in Type II, IV, V, and VI TA systems) or a non-coding RNA (in Type I and III TA systems). In Type IV TA system, the toxin protein and the antitoxin protein do not interact with each other and the antitoxin antagonizes toxin activity by stabilizing its targets [12].

Cryptic prophages are defective lysogenic prophages captured in the bacterial chromosome: either they no longer excise from the host genome or they may still excise but lose the ability to lyse the host. Hence, cryptic prophages are relatively permanent reservoirs of genes, many of which are carrying encoding restriction modification systems and TA systems [13]. In *E. coli* K-12, Type I, Type II, and Type IV TA loci have been identified in the nine cryptic prophages. The Type I TA pair RalR/RalA in the *E. coli* Rac cryptic prophage increases resistance to fosfomycin, and RalR toxin functions as a DNase [14]. The RelE toxin of Type II TA RelE/RelB in *E. coli* cryptic prophage Qin is one of the most well-studied toxins, and it functions as a sequence-specific endoribonuclease which blocks translation by differentially degrading mRNAs [15,16]. Critically, RelE increases persister cell formation [17]. Moreover, the RnlA toxin of the Type II TA system RnlA/RnlB of the *E. coli* cryptic prophage CP4-57 causes inhibition of cell growth and rapid degradation of cellular mRNAs [18].

The first recognized Type IV TA pair CbtA/CbeA was found in cryptic prophage CP4-44 in *E. coli* K-12. The toxin CbtA alters cell shape by inhibiting the polymerization of cytoskeletal proteins FtsZ and MreB via direct protein-protein interaction, without showing direct interaction with its cognate antitoxin CbeA [12]. Moreover, this TA pair has been related to resistance to norfloxacin, novobiocin, and spectinomycin [12,19,20]. The other two homologous TA loci of CbtA/CbeA also reside in prophages of *E. coli* K-12, YkfI/YafW in cryptic prophage CP4-6 and YpjF/YfjZ in cryptic prophage CP4-57 [21]. One of the most striking features of these P4-like cryptic prophages in *E. coli* is that they are pervasively mosaic, with different segments seeming to have distinct evolutionary histories [22]. The presence of three homologous TA loci in three P4-like prophages (CP4-6, CP4-44, and CP4-57) suggests these homologous fragments may evolve from one ancestor prophage. In addition to the interaction between the antitoxin and the cognate toxin (i.e., RNA-RNA, RNA-protein, protein-protein), interactions between TA systems occur at different levels. Homologous and non-homologous TA systems co-existing within a bacterial genome raises the question: how do these TA systems interact with each other and how do they impact host physiology? In this study, we first confirmed that YkfI/YafW from CP4-6 functioned as a TA pair, which is in agreement with an early study by Brown and Shaw [21], and demonstrated that YpjF/YfjZ from CP4-57 also functioned as a TA pair. We further investigated the interaction among CbtA/CbeA, YkfI/YafW, and YpjF/YfjZ. Furthermore, we investigated the physiological functions of these toxin and antitoxin genes by constructing triple deletion mutants.

## 2. Results

### 2.1. Genetic Regions Share Similarity in Three Prophages

**Figure 1:**
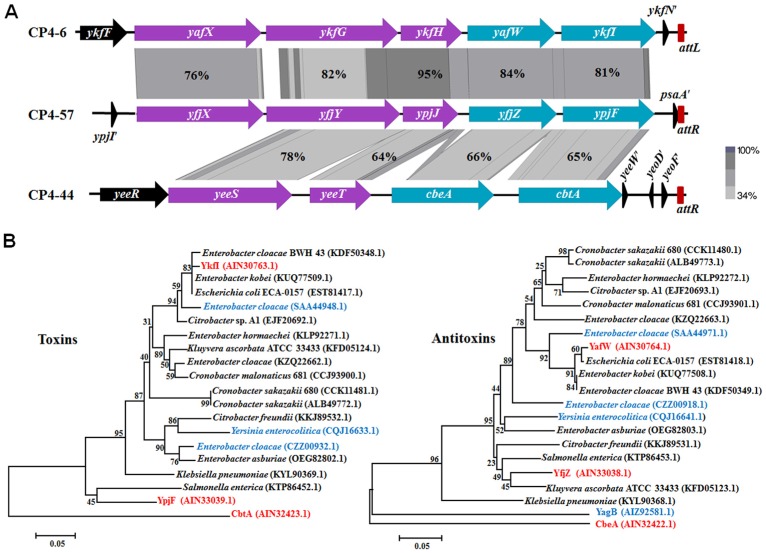
(**A**) Comparison of flanking regions containing *yafW-ykfI* in CP4-6, *yfjZ-ypjF* in CP4-57, and *cbeA-cbtA* in CP4-44. The alignment of the homologous regions was generated using Easyfig [24] based on tblastX, and the identity of the related proteins is indicated by the gradient of gray. Red bars indicate the prophage attachment sites (*attL* indicates the left attachment and *attR* indicates the right attachment site). (**B**) Evolutionary tree of the putative Type IV toxins and antitoxins. The bootstrap values in the tree refer to the substitution rate of amino acid. Three TA pairs studied here are marked in red. Non-paired toxins and antitoxins are marked in blue. The GenBank IDs of proteins used to build the phylogenetic trees are shown in the brackets.

In *E. coli* K-12, YkfI/YafW, YpjF/YfjZ, and CbtA/CbeA were initially identified as putative TA pairs, since they all consist of two neighboring genes encoding small proteins [21]. They are present in P4-like cryptic prophages of *E. coli* K-12 near the prophage attachment sites. P4-like prophages CP4-6 and CP4-57 remain excision-proficient in the *E. coli* K-12 strain, but CP4-44 lacks an integrase and has lost its ability to excise from host genome [23]. A previous study demonstrated that CbtA/CbeA consists of a Type IV TA pair [12]. Sequence analysis revealed that *yafW-ykfI* in prophage CP4-6, *yfjZ-ypjF* in prophage CP4-57, and *cbeA-cbtA* in prophage CP4-44, as well as some of the upstream regions, have similar gene arrangements, and putative gene products share medium to high sequences identity (65%–84%), which is in agreement with earlier studies [21]. High similarity in the protein sequences of the five neighboring genes was observed between CP4-6 and CP4-57 (76%–95%), and among them, four of them share medium similarity with those from CP4-44 and CP4-57 (64%–78%) (Figure 1A). The intergenic region between *ykfI* and *yafW* is 20 bases, the same as the space between *ypjF* and *yfjZ*, but there is an 88 bases intergenic region between *cbtA* and *cbeA*. Additionally, we found that *yagB* in CP4-6 encoding a protein which shares high similarity with CbeA, YafW, and YfjZ (81%–85%) (Figure 1B). However, no gene neighboring *yagB* encodes protein similar to CbtA in CP4-6 prophage, suggesting that YagB might be an orphan antitoxin. We also searched for the presence of homologs of YkfI and YafW in other sequenced bacteria. Homologs of these two proteins as a pair are found in many other *E. coli* strains, as well as in strains of *Enterobacter*, *Cronobacter*, and *Citrobacter*, and some of them are not neighbored (Figure 1B). Collectively, these results suggest a patchy distribution of putative Type IV TA loci in *E. coli* and a wide distribution of these putative Type IV TA genes in bacteria.

### 2.2. YkfI is Toxic and YafW Blocks its Toxicity

**Figure 2:**
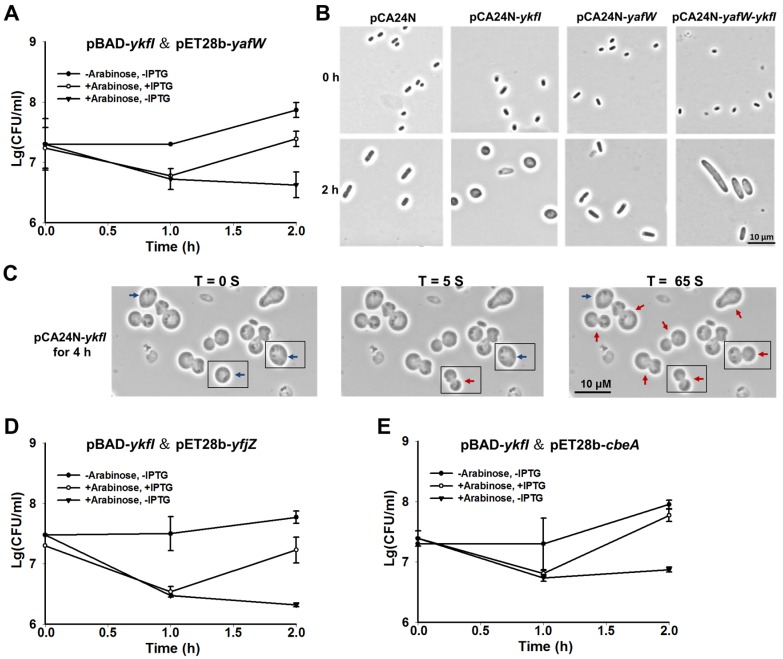
YkfI is toxic and YafW blocks its toxicity. (**A**) Cell viability of BL21 cells carrying pBAD-*ykfI* and pET28b-*yafW*. Arabinose (0.3%) was added at the beginning and isopropyl-β-d-1-thiogalactopyranoside (IPTG) (0.1 mM) was added after 1 h; (**B**) Morphology of BW25113 cells overexpressing *ykfI*, *yafW*, and *yafW-ykfI* via pCA24N-based plasmids at the time points indicated. IPTG (1 mM) was added at the beginning; (**C**) Morphology of BW25113 cells overexpressing *ykfI* via pCA24N-*ykfI* for 4 h with 1 mM IPTG. Blue arrows indicate the “lemon-shaped” cells, and red arrows indicate the “gourd-shaped” cells. Cells with apparent morphological changes within 65 seconds were all boxed; (**D**) Cell viability of BL21 cells carrying pBAD-*ykfI* and pET28b-*yfjZ*; (**E**) Cell viability of BL21 cells carrying pBAD-*ykfI* and pET28b-*cbeA*. Arabinose (0.3%) was added at the beginning, and IPTG (0.1 mM) was added after 1 h in (**D**,**E**). Mean and standard deviations are from three independent cultures.

To confirm whether YkfI and YafW constitute a TA pair like CbtA and CbeA, we first constructed a two-plasmid system in the *E. coli* BL21 host, using 0.3% arabinose to induce *ykfI* via pBAD-*ykfI*, followed by the addition of 0.1 mM of isopropyl-β-D-1-thiogalactopyranoside (IPTG) to induce *yafW* via pET28b-*yafW*. Consistent with what has been reported earlier [21], the production of YkfI was toxic, and the co-production of YafW reduced the toxic effect of YkfI, as shown by the colony forming units (CFUs), suggesting that they consist of a TA pair (Figure 2A). Furthermore, we also used pCA24N-based plasmids to test the toxicity of the two proteins in *E. coli* K-12 BW25113 host. Plasmids pCA24N-*ykfI* and pCA24N-*yafW* were obtained from the ASKA library [25]. Results showed that the overproduction of YkfI exhibited a notable decrease in cell growth, as shown by the reduction in turbidity (OD_600_) and CFUs. In contrast, overproduction of YafW did not affect cell growth (Supplementary Figure S1A,B). We further cloned the coding region of *ykfI* and *yafW* into plasmid pCA24N to construct pCA24N-*yafW-ykfI*. When YkfI and YafW were co-produced using this construct, YafW only partially neutralized the toxicity of YkfI (Supplementary Figure S1A,B). Additionally, overexpressing YkfI via pCA24N-*ykfI* caused a dramatic morphology change to the *E. coli* cells, from “rod-shaped” to “lemon-shaped” (Figure 2B). The formation of “lemon-shaped” cells has been previously reported for CbtA overproduction [19]. Additionally, we found that the prolonged production of YkfI can further cause “lemon-shaped” cells to form “gourd-shaped” cells, probably due to the loss of homoeostasis, leading to complete cell lysis within seconds or minutes (Figure 2C; Supplementary Video S1). Moreover, when YafW was co-produced with YkfI via pCA24N*-yafW-ykfI*, it inhibited the formation of “lemon-shaped” cells caused by YkfI overproduction (Figure 2B), indicating that YafW can function as the antidote for YkfI. Elongated cells were formed when YafW and YkfI were co-produced, possibly caused by the inhibition of cell division of *E. coli* cells, since these two proteins might interact with FtsZ in the same way as CbtA/CbeA [12].

Unlike the Type II and Type V TA systems we characterized previously using pCA24N-based plasmid to co-produce the toxins and the antitoxins [26,27,28], the antitoxin YafW only partially blocked the toxicity of YkfI using pCA24N*-yafW-ykfI*. As shown in Figure 1A, the three upstream genes seem to be within the same operon with *ykfI* and *yafW*, thus we checked whether these three genes can neutralize the toxicity of YkfI. The coding region of the five genes including *yafX, ykfG, ykfH, yafW*, and *ykfI* near the left attachment site (*attL*) of the prophage CP4-6 was cloned into plasmid pCA24N to make pCA24N-CP4-6-L5. When observed by comparing Supplementary Figure S1A with S1C, overexpressing the five genes together showed a similar level of toxicity as *ykfI* and *yafW* coexpressed, suggesting that the other three genes do not neutralize the toxicity of YkfI (Supplementary Figure S1C).

Different from other TA systems in *E. coli* K-12, YafW, YfjZ, and CbeA are homologous proteins. Cross-interactions between homologous toxins and antitoxins co-existing within a bacterial genome host might potentially lead to TA system redundancy, thus we tested whether the two homologous proteins of YafW can block the toxicity of YkfI. As observed, YfjZ could also neutralize the toxic effect of YkfI using 0.3% arabinose to induce *ykfI* via pBAD-*ykfI*, followed by the addition of 0.1 mM IPTG to induce *yfjZ* via pET28b-*yfjZ* in *E. coli* BL21 host (Figure 2D). Similar results were obtained for the previously reported Type IV antitoxin CbeA, which can also neutralize the toxic effect of YkfI (Figure 2E).

### 2.3. YpjF is Toxic and YfjZ Blocks its Toxicity

**Figure 3:**
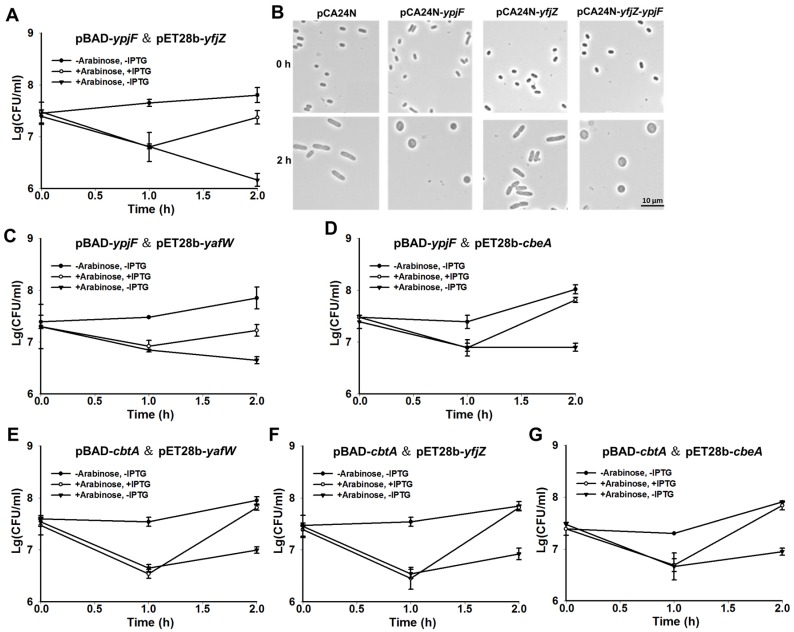
YpjF is toxic and YfjZ blocks its toxicity. (**A**) Cell viability of BL21 cells carrying pBAD-*ypjF* and pET28b-*yfjZ*. Arabinose (0.3%) was added at the beginning, and IPTG (0.1 mM) was added 1 h later; (**B**) Morphology of BW25113 cells overexpressing *ypjF*, *yfjZ*, and *yfjZ-ypjF* via pCA24N-based plasmids at the time points indicated. IPTG (1 mM) was added at the beginning; (**C**) Cell viability of BL21 cells carrying pBAD-*ypjF* and pET28b-*yafW*; (**D**) Cell viability of BL21 cells carrying pBAD-*ypjF* and pET28b-*cbeA*; (**E**) Cell viability of BL21 cells carrying pBAD-*cbtA* and pET28b-*yafW*; (**F**) Cell viability of BL21 cells carrying pBAD-*cbtA* and pET28b-*yfjZ*. (**G**) Cell viability of BL21 cells carrying pBAD-*cbtA* and pET28b-*cbeA*. Arabinose (0.3%) was added at the beginning, and IPTG (0.1 mM) was added after 1 h in (**C**–**G**). Mean and standard deviations are from three independent cultures.

To test whether YpjF and YfjZ constitute a TA pair, we constructed a two-plasmid system by using 0.3% arabinose to induce *ypjF* via pBAD-*ypjF*, followed by the addition of 0.1 mM IPTG to induce *yfjZ* via pET28b-*yfjZ* in the *E. coli* BL21 host. The production of YpjF was toxic and the co-production of YfjZ reduced the toxic effect of YpjF, suggesting that they consist of a TA pair (Figure 3A). Furthermore, we also used pCA24N-based plasmids to test the toxicity of the two proteins in BW25113 host. Results showed that overproduction of YpjF exhibited a notable decrease in cell growth. In contrast, overproduction of YfjZ did not affect cell growth (Supplementary Figure S1D,E). We further cloned the coding regions of *yfjZ* and *ypjF* into plasmid pCA24N to construct pCA24N-*yfjZ-ypjF*. When YfjZ and YpjF were co-produced using this construct, YfjZ only slightly reduced the toxicity of YpjF (Supplementary Figure S1D,E). Additionally, overexpressing *ypjF* via pCA24N-*ypjF* caused a dramatic morphology change to the *E. coli* cells, from “rod-shaped” to “lemon-shaped” (Figure 3B). Unlike YafW, YfjZ was unable to prevent the formation of “lemon-shaped” cells caused by YpjF overproduction (Figure 3B). Next, we cloned the coding region of five genes including *yfjX, yfjY, ypjJ, yfjZ*, and *ypjF* near the right attachment site of the prophage (*attR*) into plasmid pCA24N to make pCA24N-CP4-57-R5. Overexpressing the five genes still showed toxicity (Supplementary Figure S1F) and cannot prevent the formation of “lemon-shaped” cells.

Furthermore, we tested whether the two homologous proteins of YfjZ can block the toxicity of YpjF using the two-plasmid system. We found that YafW and CbeA could also neutralize the toxic effect of YpjF (Figure 3C,D). Similarly, the two homologous proteins of CbeA, YafW and YfjZ, can block the toxicity of CbtA using the two-plasmid system (Figure 3E–G).

### 2.4. YkfI and YpjF Interact with FtsZ

**Figure 4:**
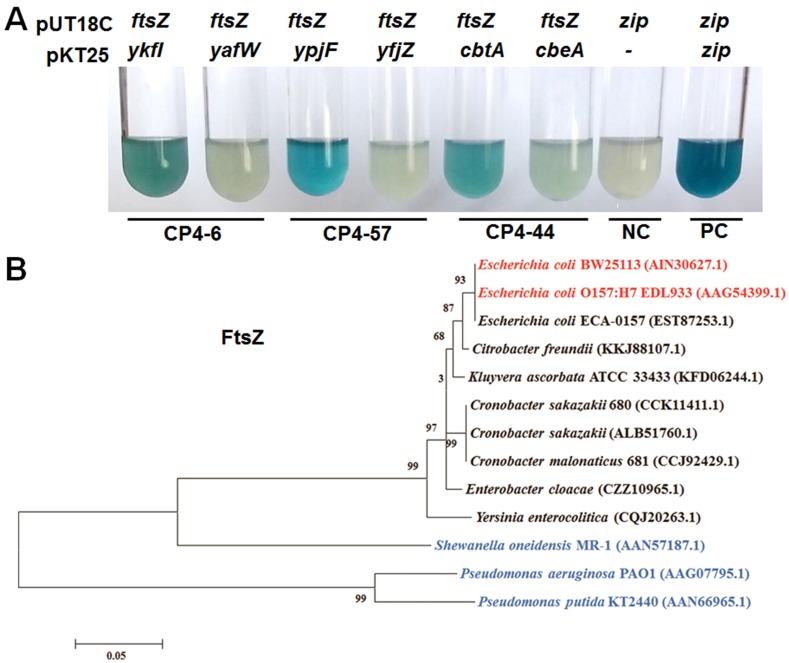
(**A**) YkfI and YpjF interact with FtsZ as shown by Cya-based bacterial two-hybrid (BATCH) assay based on the physical interaction of the T18 and T25 catalytic domain. NC indicates negative control of pUT18C vector with a DNA insert from a 35-aa-long leucine zipper (*zip*) and pKT25 with no insert, while the positive control (PC) was *zip* inserted in both pUT18C and pKT25 vectors. Three independent cultures of each strain were tested and only representative images are shown; (**B**) Evolutionary tree of the FtsZ protein. Error bar represents substitution rate of amino acid. The GenBank IDs of proteins used to build the phylogenetic trees are shown in the brackets. Strains that form “lemon-shaped” cells by the overexpressing of toxins are marked in red, and strains that do not form “lemon-shaped” cells by the overexpressing of toxins are marked in blue.

A previous study reported that, unlike Type II TA systems in which both the toxin and antitoxin are proteins and form a complex, the Type IV toxin protein CbtA and Type IV antitoxin protein CbeA did not form a complex in vivo [12]. The CbtA toxin inhibits FtsZ and MreB in *E. coli*, two essential proteins involved in the cytoskeleton and cell division, respectively [19]. The antitoxin protein CbeA does not interact with CbtA directly, but promotes the bundling of FtsZ and MreB [12]. We found that the toxins YkfI and YpjF also caused “lemon-shaped” cell formation, suggesting that they might also inhibit the polymerization of the cytoskeleton proteins. Thus, we used BACTH (Bacterial Adenylate Cyclase Two-Hybrid) assay to test whether these proteins interact with FtsZ and MreB based on the physical interaction of the T18 and T25 catalytic domains [29]. The coding region of *ftsZ* or *mreB* was fused to the C terminus of T18 fragment and cloned into pUT18C to create the in-frame translational fusions of a T18 catalytic domain. Similarly, the six genes from the three prophages were fused to T25 fragment and cloned to pKT25 to create the in-frame translational fusion of a T25 catalytic domain. A pUT18C vector with DNA insert from a 35-aa-long leucine zipper and a pKT25 vector with no insert were used as a negative control, and pUT18C and pKT25 vectors with DNA from a 35-aa-long leucine zipper inserted were used as a positive control. Our BACTH results verified that CbtA interacted with FtsZ as expected. Moreover, YkfI and YpjF also interacted with FtsZ (Figure 4A). However, no interaction was detected between these proteins (toxins and antitoxins) and MreB using BATCH assay (data not shown). Results of β-galactosidase activity assays showed that the affinity of toxin proteins with FtsZ is much higher than those of the antitoxin proteins (Supplementary Figure S2).

Since these Type IV toxins are found in prophages in *E. coli* and their target is FtsZ, we further performed experiments to test the toxicity of the ectopic expressing of these proteins in other bacteria. In enterohaemorrhagic *E. coli* O157:H7 (EDL933), the expression of either of these toxins greatly inhibited cell growth and formed “lemon-shaped” cells (Supplementary Figure S3). However, neither of the toxins caused the formation of “lemon-shaped” cells or resulted in severe growth inhibition in other clinically or ecologically important Gammaproteobacteria strains such as *Pseudomonas aeruginosa* PAO1, *Pseudomonas putida* KT2440, and *Shewanella oneidensis* MR-1 (Supplementary Figure S4). Overproduction of the three toxins in *P. putida* and *S. oneidensis* caused minor filamentous growth, possibly due to ectopic expression of proteins in these hosts. To explain the differences in toxicity of toxins in different hosts, we compared the protein sequences of FtsZ of different hosts. As expected, EDL933 and BW25113 share 100% identity in the amino acid sequence of FtsZ, but the other tested strains share low sequence similarity of FtsZ with *E. coli* K-12 BW25113 (Figure 4B), suggesting a lack of direct interaction between these toxins and FtsZ in *Pseudomonas* and *Shewanella*.

### 2.5. Type IV TAs Influence Stress Response and Biofilm Formation

**Figure 5:**
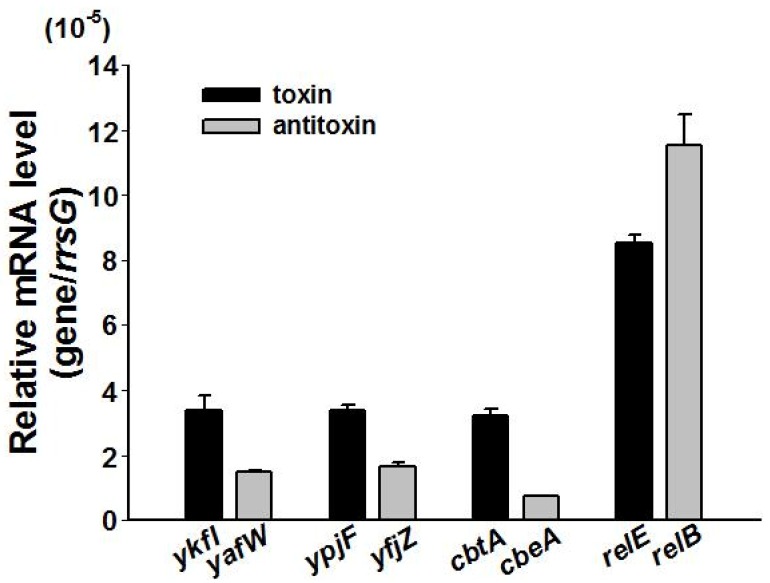
The relative mRNA levels of the three toxins and the three antitoxins as compared to Type II toxin *relE* and antitoxin *relB* in BW25113 wild type measured at OD ~ 1.0. The housekeeping gene *rrsG* was used to normalize the gene expression data. Data are from three independent cultures, and standard deviations are shown.

By deleting all nine prophages in *E. coli* K-12 BW25113, we previously found that the cryptic prophages are beneficial for withstanding oxidative and acid stresses, and for influencing the resistance to antibiotics [23]. To explore the physiological function of three TA loci, we obtained single deletion mutants from the Keio Collection [30], and constructed multiple deletion mutants using P1 transduction [31]. All of the single and multiple deletion mutants were confirmed by PCR followed by DNA sequencing (Supplementary Figure S5). Deletion of all the three toxin genes or deletion of all of the three antitoxin genes did not affect cell growth (Supplementary Figure S6). The mRNA levels of antitoxin and toxin genes were measured via quantitative real-time reverse-transcription PCR (qRT-PCR) in the exponentially growing cells in the wild type strain. Results showed that these three toxins and antitoxins were transcribed at relatively low levels as compared to the Type II TA toxin gene *relE* and antitoxin gene *relB* (Figure 5). In Type II and Type IV TA systems, both the toxin and antitoxin are proteins. In typical Type II TA systems, the antitoxin autoregulates the TA operon via its DNA binding ability [32]. Thus, we also measured the expression level of the TA operon in the toxin or antitoxin deleted strains. Results showed that the mRNA level of the toxin was similar in the antitoxin deleted strain and in the wild type strain (Supplementary Figure S7A), and the mRNA level of the antitoxin was similar in the toxin deleted strain and in the wild type strain (Supplementary Figure S7B). These results suggest that neither the toxin nor the antitoxin autoregulates the Type IV TA operon.

**Figure 6:**
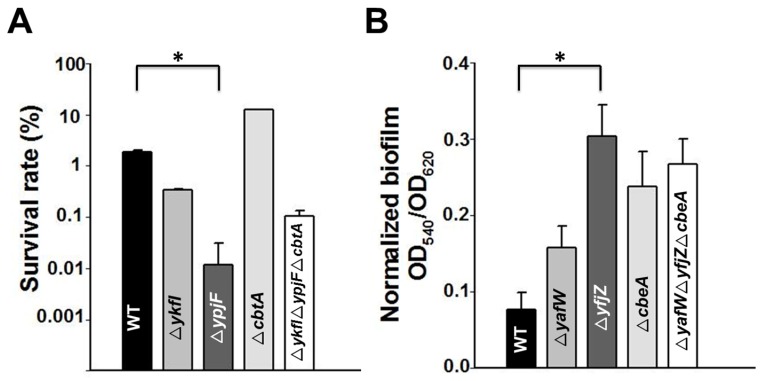
(**A**) Cell viability of the single deletion and multiple deletion strains as compared to the BW25113 wild type strain (WT) in the presence of oxidative stress (30 mM H_2_O_2_ for 10 min); (**B**) Normalized biofilm formation of the single deletion and multiple deletion strains as compared to the WT measured at 6 h in Luria–Bertani (LB) medium. Error bars indicate standard deviation. Asterisks represent statistically significant differences using Kruskal-Wallis test (*p* < 0.01 was shown in *). Mean and standard deviations are from three independent cultures.

To probe whether the toxin and antitoxin genes in these three prophages contribute to stress response, we tested the resistance of these deletion mutant strains to oxidative stress (30 mM H_2_O_2_ for 10 min). Results showed that deletion of all of the three toxin genes together (deletion mutant Δ*ykfI*Δ*ypjF*Δ*cbtA*) decreased resistance to oxidative stress, and deletion of toxin gene *ypjF* reduced resistance to oxidative stress by approximately 100-fold (Figure 6A). However, deletion of the antitoxin genes did not affect resistance to oxidative stress (data not shown). Biofilm formation was also measured for these deletion mutants using a 96-well polystyrene plate assay. The deletion of either one or three antitoxin genes increased early biofilm formation 2~4-fold (Figure 6B). However, in the triple toxin deletion strain, there was only a minor increase of biofilm formation (Supplementary Figure S8). Moreover, resistance to six different antibiotics of these strains was also measured using a minimum inhibitory concentration (MIC) test. Unexpectedly, we found that the deletion of the *cbtA* and *cbeA* genes slightly increased resistance to kanamycin and ciprofloxacin, while no differences were found for polymyxin B and tetracycline (Supplementary Table S2). However, we could not rule out the possibility that there are other side mutations that might lead to the phenotypes observed here.

## 3. Discussion

Among the six different types of TA systems identified so far, Type II, IV, V, and VI TAs all consist of a toxin gene neighbored by an antitoxin gene. One unique feature of Type IV TA system is that the toxin and antitoxin do not show a direct interaction with each other [12]. By studying two homologous loci of previous characterized Type IV TA CbtA/CbeA in prophages of CP4-6 and CP4-57 in *E. coli* K-12 BW25113, we demonstrate that all three TA loci shared similar features. We demonstrated that: (i) YkfI, YpjF, and CbtA are potent toxins and they all interact with FtsZ; (ii) antitoxins YafW, YfjZ, and CbeA can block the toxicity of the toxins YkfI, YpjF, and CbtA; and (iii) neither the toxin nor the antitoxin autoregulates the TA operon. In most TA systems, inhibition of antitoxin synthesis is a crucial step for TA activation. Due to the different features of the Type IV TAs, the manner of activation for these Type IV toxins remains unknown. Future studies are needed to determine what the external signals are and what genetic pathways lead to the activation of Type IV toxin.

In many cases, TA systems are clustered and closely linked to mobile genetic elements [33]. Functions of TA systems in host chromosome and mobile genetic elements appear to be quite different. Roles of TAs in plasmids have been extensively studied [34]; however, roles of TA in other mobile genetic elements have just started to be revealed. Integrative and conjugative elements (ICEs) are a diverse group of mobile genetic elements found in many bacteria, and a Type II TA pair MosT/MosA from ICE in *Vibrio cholera* was found to promote its maintenance [9]. Type II TA systems are extremely common in superintegrons in *Vibrio* genomes, and these TAs play roles in the stabilization of these superintegrons [35]. Another characterized Type IV TA system, AbiE, which is widespread in bacterial and archaeal genomes and in mobile genetic elements, provides phages resistance and enable stabilization [36]. Previous work by Brown and Shawn showed that YpjF, YkfI, and CbtA are toxic proteins, and YafW and CbeA can block the toxicity of YkfI and CbtA [21]. Here, we further tested the interaction between these homologous toxins and antitoxins in the cryptic prophages of *E. coli*, and found that YafW, YfjZ and CbeA can function as the antitoxin for these three homologous toxins. Interactions among different TA systems have been previously investigated on different levels. Co-occurrence of these homologous TA loci within a bacterial genome still remains enigmatic and needs further investigation.

Brown and Shaw have demonstrated that untagged YpjF and C-terminal His-tagged YpjF did not cause growth inhibition, while N-terminal FLAG-tagged YpjF inhibited growth [21]. In this study, we show that both untagged YpjF produced via pBAD-*ypjF* and pHGE-*ypjF* and N-terminal His-tagged YpjF produced via pCA24N-*ypjF* inhibited growth and caused morphological changes. The observed differences in YpjF toxicity might be caused by different amounts of YpjF produced using different vectors or the tagging at C terminus might affect the toxicity of YpjF. In some TA systems, the antitoxin cannot be deleted from the chromosome due to the de-repression of toxin production in the absence of the cognate antitoxin [28]. The ability to delete the Type IV antitoxin in this study might be due to the low expression of Type IV toxin gene and/or the lack of autoregulation of Type IV TA operon by the antitoxin under normal growing conditions.

Several cell division inhibitor genes are found in cryptic prophages of *E. coli.* Lambdoid *kil* genes can inhibit cell division to maintain synchrony with the host in its lysogenic state [37], and lambdoid cryptic prophage Rac has a functional *kilR* gene interacting with FtsZ and ZipA to prevent FtsZ assembly into a division-competent ring structure [38]. DicB from prophage Qin can rapidly cease cell division by targeting FtsZ when it is induced under stress [39] or through interaction with MinC [40]. In addition, lambdoid cryptic prophage e14 has a functional cell division inhibitor gene *sfiC* that is possibly encoded by *ymfL* or *ymfM* [41]. DicF is a small RNA in prophage Qin in the genomes of many *E. coli* strains including *E. coli* K-12, and a recent study showed that DicF inhibits cell division via direct base pairing with *ftsZ* mRNA to repress translation and prevent new synthesis of the bacterial tubulin homolog FtsZ [42]. Previously identified Type IV toxin CbtA inhibits cell division by targeting FtsZ and MreB [19]. In this study, we demonstrated that YkfI and YpjF interact with FtsZ with the same affinity as CbtA, leading to a similar morphology change. Additionally, Type IV antitoxin CbeA was found to directly interact with MreB and FtsZ [12]. The physiological role of these cell division inhibitor genes in cryptic prophages has not been fully elucidated. We have previously showed that KilR from prophage Rac and DicB from prophage Qin increased resistance to nalidixic acid and azlocillin [23]. YkfI/YafW is related to the resistance to bacteriocin colicin E3 [43], and YpjF/YfjZ is related to resistance to novobiocin [44]. Moreover, CbtA/CbeA pair has related to resistance to norfloxacin, novobiocin, and spectinomycin [12,19,20]. Here we found that YpjF increased resistance to oxidative stress. These results collectively show that prophage genes somehow give the host selective advantages at different stress conditions. Additionlly, toxins CbtA, YkfI, and YpjF cause cell death by interacting with FtsZ in *E. coli* strains, but not in *Pseudomonas* strains, suggesting that they might be considered as promising targets for the antibacterial drugs and their potential in controlling infectious disease.

## 4. Materials and Methods

### 4.1. Bacterial Strains, Plasmids, and Growth Conditions

*E. coli* K-12 BW25113 and the isogenic strains were grown in Luria–Bertani (LB) medium at 37 °C. Chloramphenicol (30 μg/mL) and carbenicillin (100 μg/mL) were used for maintaining pCA24N and pHERD20T plasmids, respectively. Ampicillin (100 μg/mL) was used for maintaining the pKT25 or pBAD plasmid, and kanamycin (50 μg/mL) was used for pUT18C, pHGE, or pET28b plasmid. For the growth and CFU assays, isopropyl-β-D-1-thiogalactopyranoside (IPTG) and/or arabinose were added as the inducer. For the stress assays, 30 mM H_2_O_2_ was added to exponentially growing cells (OD_600_ ~ 0.8), and cell viability was determined by serial dilutions on LB agar plates. Determination of the minimum inhibitory concentration (MIC) was performed using antibiotic sensitive plates (Biofosun Biotech, Shanghai, China) with a specific antibiotic concentration gradient and incubated at 37 °C for 18–22 h.

### 4.2. Plasmids Construction

The DNA regions containing *yafW-ykfI*, *yfjZ-ypjF*, five genes in CP4-6 (Supplementary Figure S1C) and five genes in CP4-57 (Supplementary Figure S1F) were amplified from BW25113 using primer pairs listed in Supplementary Table S3. The expected PCR products were digested with *Sal*I/*Hin*dIII (New England Biolabs, NEB) and cloned into the corresponding sites of pCA24N, generating pCA24N*-yafW-ykfI*, pCA24N*-yfjZ-ypjF*, pCA24N-CP4-6-L5, and pCA24N-CP4-57-R5. The constructs were confirmed by PCR followed by DNA sequencing using primers pCA24N-F/-R. The other recombinant plasmids of pBAD, pHGE, pUT18C, and pHERD20T were constructed following similar steps. The detailed information of primer pairs used for PCR amplification, restriction enzyme sites used in digestion of the PCR products, and the primers used for PCR sequencing are shown in Supplementary Table S3. The recombinant plasmids pKT25-*ftsZ*, pKT25-*mreB*, pET28b-*yafW*, pET28b-*yfjZ*, and pET28b-*cbeA* were constructed with ClonExpress^TM^ II One Step Cloning Kit (Vazyme Biotech, Piscataway, NJ, USA), and the correct constructs were confirmed by PCR using primer pairs pKT25-F/-R or T7-F/-R.

### 4.3. Microscopic Observation

For microscopy sample preparation, overnight cultures were diluted to OD_600_ ~0.05 in fresh LB. Strains were induced with 1 mM IPTG and cultured at 37 °C with shaking. Cells were washed three times with PBS (pH 7.4) before observation, and examined using a ZEISS AX10 microscope (Carl Zeiss AG, Oberkochen, Germany) at different time points.

### 4.4. BACTH Assay

Plasmids used for the BACTH assay (Supplementary Table S1) were transformed into *E. coli* BTH101 (BACTH System Kit, Euromedex, Souffelweyersheim, France) [29]. Transformants were plated onto LB medium containing 100 μg/mL ampicillin and 50 μg/mL kanamycin, 200 μg/mL 5-bromo-4-chloro-3-indolyl-β-d-galactopyranoside (X-Gal), and 1 mM IPTG. Strains were induced with 1 mM IPTG for 3 h after dilution of OD_600_ to ~0.05, and OD_600_ was measured. Cultures were diluted 1:5 into PM2 (70 mM Na_2_HPO_4_·12H_2_O, 30 mM NaH_2_PO_4_·H_2_O, 1 mM MgSO_4_, and 0.2 mM MnSO_4_, pH 7.0) [45]. To permeabilize cells, 30 μL of toluene and 35 μL of a 0.1% SDS solution were added to 2.5 mL of bacterial suspension. The tubes were vortexed for 10 s and incubated at 37 °C for 45 min for evaporation of toluene. For the enzymatic reaction, 250 μL of permeabilized cells was added to PM2 supplemented with β-mercaptoethanol (final concentration, 100 mM) to a final volume of 1 mL. The reaction was started by adding 250 μL of 4 mg/ mL 1 *O*-nitrophenol-galactoside in PM2. Na_2_CO_3_ was added to stop the reaction and OD_420_ was measured [46]. The β-galactosidase activity, *A* (in units per milliliter), was calculated according to the following equation: *A =* 200 × ((OD_420_ of the culture − OD_420_ in the control tube)/minutes of incubation ×dilution factor)/ OD_600_.

### 4.5. Biofilm Assay

Normalized biofilm formation was assayed in 96-well polystyrene plates (Corning Costar, Cambridge, MA, USA) in LB medium at 30 °C with crystal violet staining [47]. To remove growth effects, biofilm formation was normalized by dividing bacterial growth for each strain (OD_540_/OD_620_).

### 4.6. RNA Isolation and Quantitative Real-Time Reverse-Transcription PCR (qRT-PCR)

To compare the expression of toxin and antitoxin genes in the corresponding antitoxin and toxin deletion strains with the BW25113 wild type strain, a qRT-PCR assay was performed. Total RNAs were isolated using the QIAGEN RNase Mini kit (Valencia, CA, USA), as described previously [48]. The cDNA synthesis was conducted using the reverse transcription system according to the instruction and operation manual (Promega, Madison, WI, USA). The qRT-PCR reaction was performed using the ChamQ SYBR qPCR Master Mix (Vazyme Biotech, Piscataway, NJ, USA). The housekeeping gene *rrsG* (16S rRNA gene) was used to normalize the gene expression data. The primers used for the quantification of the toxin genes and the antitoxin genes were shown in Supplementary Table S3.

### 4.7. Construction of Multiple Gene Deletion Mutants

Multiple deletion mutants of *E. coli* K-12 BW25113 were constructed using P1 transduction based on the single deletion mutants available in Keio collection [27,28]. For example, to obtain the triple mutant Δ*ykfI*Δ*ypjF*Δ*cbtA*, P1 transduction was first used to transfer the Δ*ypjF* Km^R^ mutation to Δ*ykfI* to obtain strain Δ*ykfI*Δ*ypjF* Km^R^. The kanamycin resistance cassette from the newly constructed multiple deletion strain Δ*ykfI*Δ*ypjF* Km^R^ was removed with the helper plasmid pCP20 [49], and this strain was used as a recipient for P1 transduction to transfer Δ*cbtA* Km^R^ to obtain Δ*ykfI*Δ*ypjF*Δ*cbtA* Km^R^. The triple deletion mutant Δ*ykfI*Δ*ypjF*Δ*cbtA* was obtained after Km^R^ was removed. Similarly, the triple deletion mutant Δ*yafW*Δ*yfjZ*Δ*cbeA* was obtained following the same procedure. Single and triple deletion mutants were verified by PCR followed by DNA sequencing using primers listed in Supplementary Table S2.

## Supplementary Materials

The following are available online at www.mdpi.com/2072-6651/9/3/77/s1, Figure S1: Cell growth, cell viability, and toxic plates of BW25113 cells overexpressing toxins and antitoxins via pCA24N-based plasmids, Figure S2: BACTH assay, Figure S3: The expression of toxin YkfI, YpjF, and CbtA can produce “lemon-shaped” cells in *Escherichia coli* O157:H7 (EDL933), Figure S4: Ectopic overproduction of toxin YkfI, YpjF, or CbtA cannot produce “lemon-shaped” cells in (A) *Pseudomonas aeruginosa* PAO1, (B) *Pseudomonas putida* KT2440, and (C) *Shewallena oneidensis* MR-1 hosts, Figure S5: PCR verification of deletion strains, Figure S6: Deletion of all the three toxin genes or the three antitoxin genes did not affect cell growth (A) and cell viability (B), Figure S7: The mRNA expression of toxin or antitoxin was not changed in the corresponding antitoxin or toxin deleted strains compared with the wild type strain, Figure S8: Biofilm formation of the single toxin deletion and triple toxins deletion strains as compared to the BW25113 wild type strain (WT) measured at 6 h in LB medium, Table S1: Strains and plasmids used in this study, Table S2: Minimum inhibitory concentration (MIC) value for the BW25113 wild type strain and the deletion mutant strains, Table S3: Primers used in this study, Video S1: Over-production of toxin YkfI cause “lemon-shaped” cells to further form “gourd-shaped” cells.
