# Supplementary material for: Interaction of Type IV Toxin/Antitoxin Systems in Cryptic Prophages of Escherichia coli K-12
Source: Toxins (Basel). 2017 Mar 1;9(3):77. doi: 10.3390/toxins9030077 (PMC5371832; doi:10.3390/toxins9030077)
Supplement: Supplementary file 1 [file toxins-09-00077-s001.zip › toxins-168664 supplementary conversion.pdf]

Supplementary Materials: Interaction of Type IV Toxin/Antitoxin Systems in Cryptic Prophages of *Escherichia coli* K-12

Zhongling Wen, Pengxia Wang, Chenglong Sun, Yunxue Guo and Xiaoxue Wang

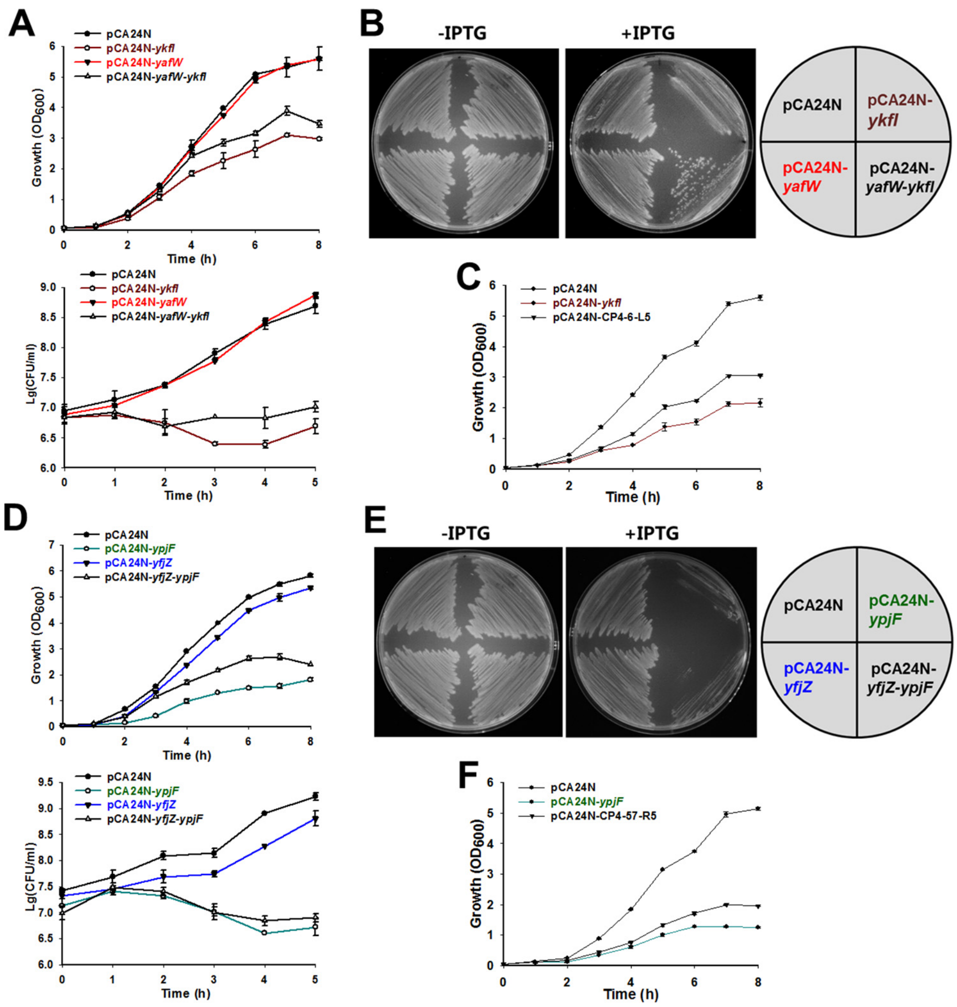

Figure S1. Cell growth, cell viability and toxic plates of BW25113 cells overexpressing toxins and antitoxins via pCA24N-based plasmids. (**A**) Cell growth (upper panel) and cell viability (lower panel) of BW25113 cells overexpressing *ykfI*, *yafW* and *yafW-ykfI*. (**B**) Strains from (**A**) were streaked onto LB plates with or without 1 mM IPTG, and were incubated for 16 h. (**C**) Cell growth of BW25113 cells overexpressing *ykfI* and the five genes near the left attachment site of CP4-6 (CP4-6-L5). (**D**) Cell growth (upper panel) and cell viability (lower panel) of BW25113 cells overexpressing *ypjF*, *yfjZ* and *yfjZ-ypjF*. (**E**) Strains from (**D**) were streaked onto LB plates with or without 1 mM IPTG, and were incubated for 16 h. (**F**) Cell growth of BW25113 overexpressing *ypjF* and the five genes near the right attachment site of CP4-57 (CP4-57-R5). IPTG (1 mM) was added at the beginning in A, C, D and F. Data are from two independent cultures, and standard deviations are shown.

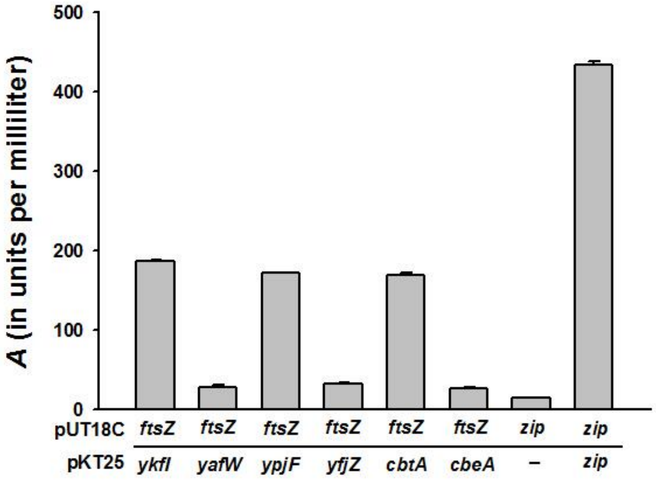

Figure S2. BACTH assay. The beta-gal enzymatic activity, A (in units per milliliter), was calculated according to the following equation: A = 200× ((OD_{420} of the culture - OD_{420} in the control tube)/minutes of incubation ×dilution factor)/ OD_{600}. Data are from three independent cultures, and standard deviations are as shown.

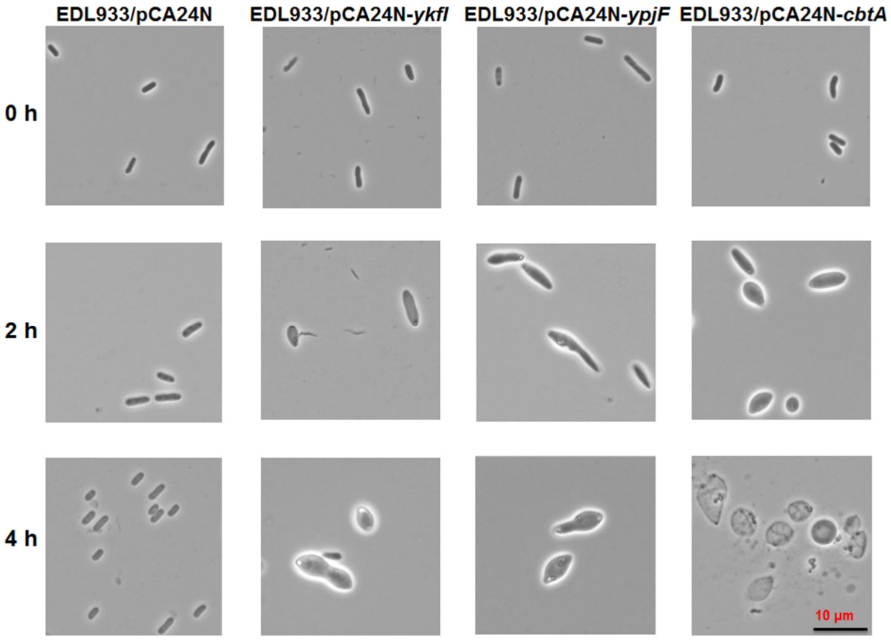

Figure S3. The expression of toxin YkfI, YpjF and CbtA can produce "lemon-shaped" cells in *Escherichia coli* O157:H7 (EDL933).

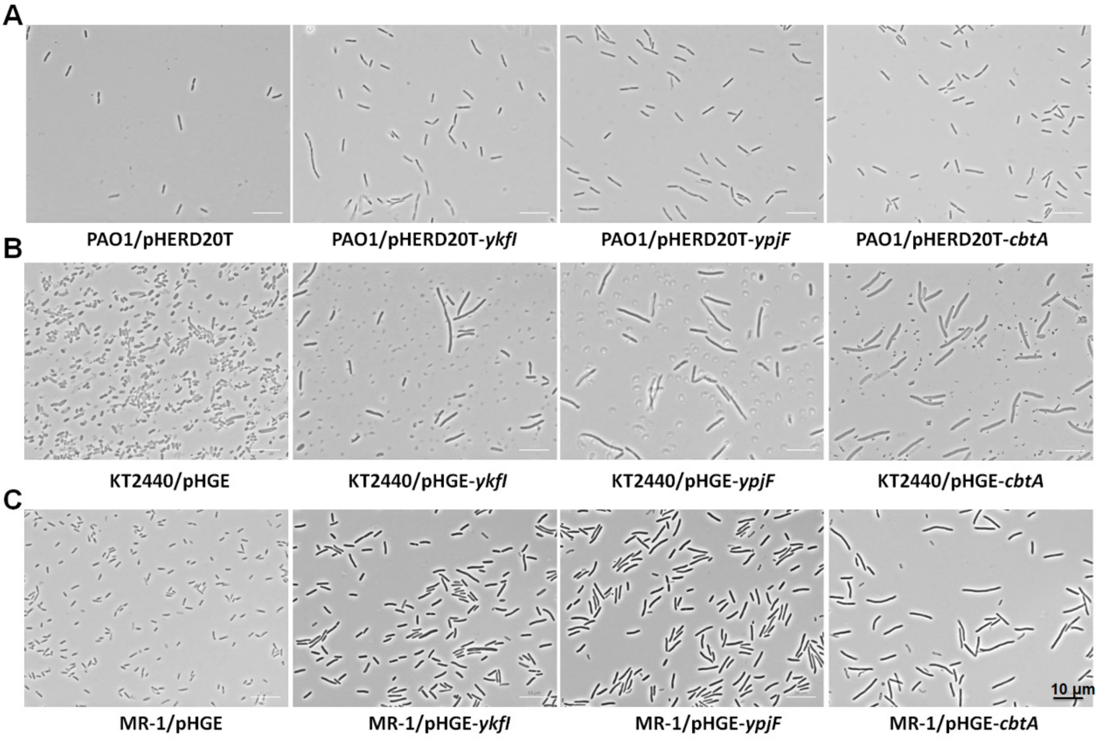

Figure S4. Ectopic overproduction of toxin YkfI, YpjF or CbtA can not produce "lemon-shaped" cells in (**A**) *Pseudomonas aeruginosa* PAO1, (**B**) *Pseudomonas putida* KT2440 and (**C**) *Shewallena oneidensis* MR-1 hosts.

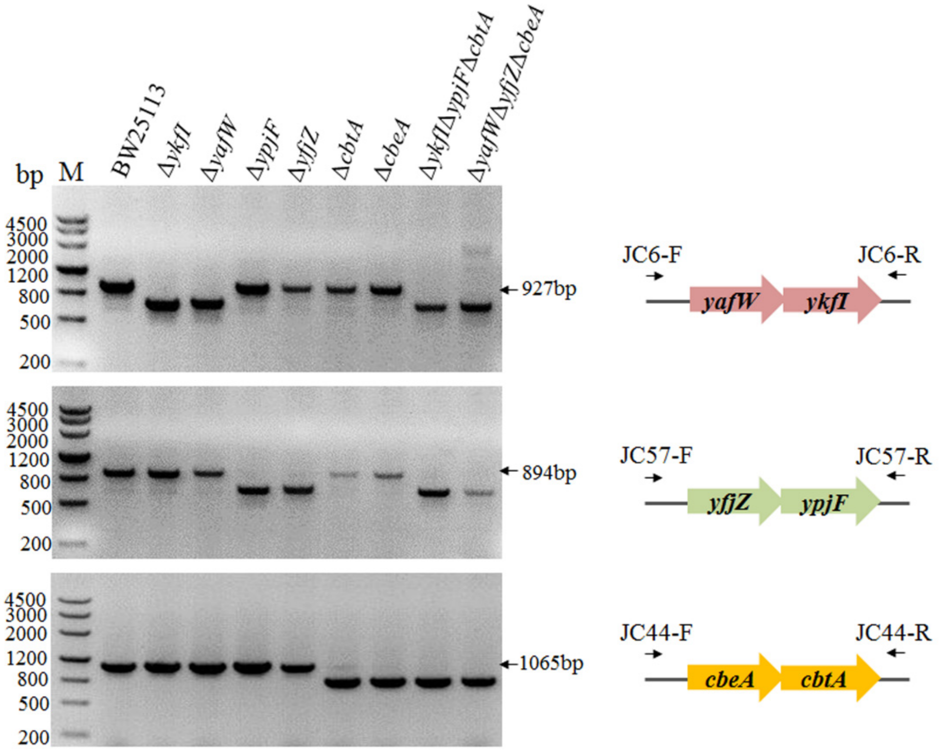

Figure S5. PCR verification of deletion strains. The PCR was amplified using specific primers JC6-F/-R, JC57-F/-R and JC44-F/-R (Supplementary Table S3) flanking *yafW-ykfI*, *yfjZ-ypjZ* and *cbeA-cbtA*. DNA from the deletion strains was used as the PCR templates, and BW25113 was used as a positive control. The arrows on the right side of the gels indicate the size of the PCR products using the DNA of BW25113 as templates.

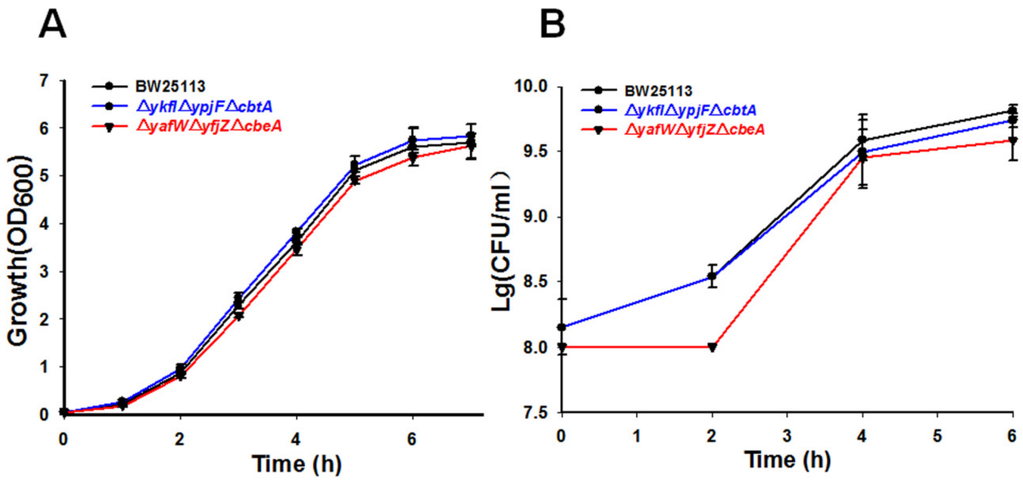

Figure S6. Deletion of all the three toxin genes or the three antitoxin genes did not affect cell growth (**A**) and cell viability (**B**). Data are from two independent cultures, and standard deviations are shown in A and B.

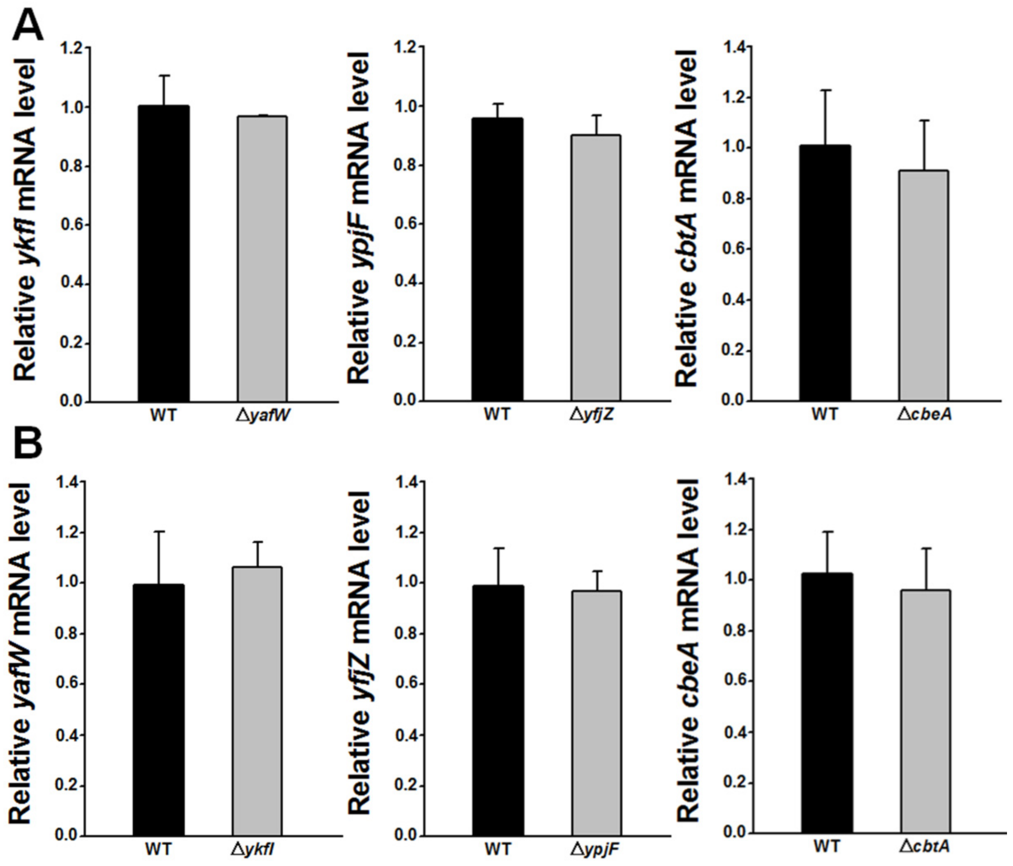

Figure S7. The mRNA expression of toxin or antitoxin was not changed in the corresponding antitoxin or toxin deleted strains compared with the wild type strain. (**A**) The relative toxins mRNA level has no difference in the BW25113 wild type strain and antitoxin deletion strains. (**B**) The relative antitoxins mRNA level has no difference in the BW25113 wild type strain and toxin deletion strains. Data are from two independent cultures, and standard deviations are shown in A and B.

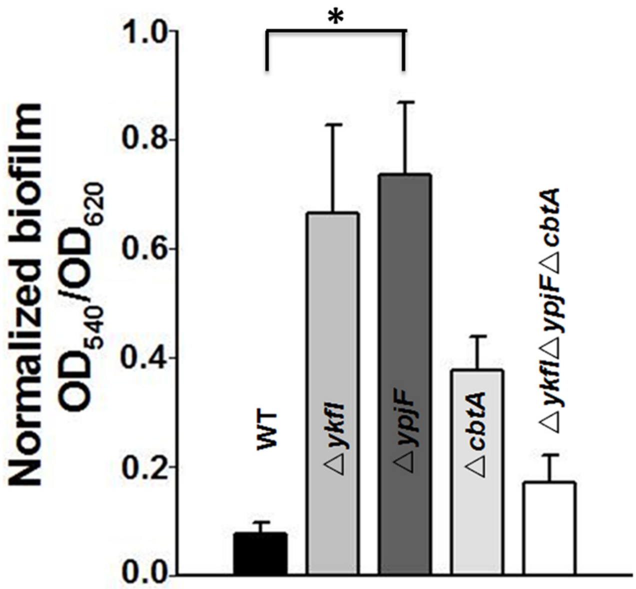

Figure S8. Biofilm formation of the single toxin deletion and triple toxins deletion strains as compared to the BW25113 wild type strain (WT) measured at 6 h in LB medium. Asterisks represent statistically significant differences using Kruskal-Wallis test ($p < 0.01$ was shown in *). Mean and standard deviations are from three independent cultures.

Table S1. Strains and plasmids used in this study.

Strains/Plasmids	Description	Reference or Source
	E. coli strains	
K-12 BW25113	*lacI*^{q} *rrnB*$_{T14}$ Δ*lacZ*$_{WJ16}$ *hsdR514* Δ*araBA*$_{\Delta AH33}$ Δ*rhaBAD*$_{LD78}$	[1]
BTH 101	*F*$^{-}$, *cya-99*, *araD139*, *galE15*, *galK16*, *rpsL1* (*Str*^{r}), *hsdR2*, *mcrA1*, *mcrB1*	Euromedex Kit
EDL933	Wild type	[2]
BW25113 *ykfI*	BW25113 Δ*ykfI* Ω Km^{R}	[1]
BW25113 *yafW*	BW25113 Δ*yafW* Ω Km^{R}	[1]
BW25113 *ypjF*	BW25113 Δ*ypjF* Ω Km^{R}	[1]
BW25113 *yfjZ*	BW25113 Δ*yfjZ* Ω Km^{R}	[1]
BW25113 *cbtA*	BW25113 Δ*cbtA* Ω Km^{R}	[1]
BW25113 *cbeA*	BW25113 Δ*cbeA* Ω Km^{R}	[1]
Δ*ykfI*	BW25113 Δ*ykfI* with Km^{R} removed	This study
Δ*yafW*	BW25113 Δ*yafW* with Km^{R} removed	This study
Δ*ypjF*	BW25113 Δ*ypjF* with Km^{R} removed	This study
Δ*yfjZ*	BW25113 Δ*yfjZ* with Km^{R} removed	This study
Δ*cbtA*	BW25113 Δ*cbtA* with Km^{R} removed	This study
Δ*cbeA*	BW25113 Δ*cbeA* with Km^{R} removed	This study
Δ*ykfI*Δ*ypjF*Δ*cbtA*	BW25113 Δ*ykfI*Δ*ypjF*Δ*cbtA* with Km^{R} removed	This study
Δ*yafW*Δ*yfjZ*Δ*cbeA*	BW25113 Δ*yafW*Δ*yfjZ*Δ*cbeA* with Km^{R} removed	This study
	Other strains	
PAO1	*Pseudomonas aeruginosa* PAO1, wild type	[3]
KT2440	*Pseudomonas putida* KT2440, wild type	ATCC 12633
MR-1	*Shewallena oneidensis* MR-1, wild type	[4]
	Plasmids	
pCP20	Amp^{R} and Cm^{R}; temperature sensitive replication, thermal induction of FLP recombinase synthesis	[5]
pCA24N	Cm^{R}; lacI^{q}, IPTG inducible expression vector	[6]
pCA24N-*ykfI*	Cm^{R}; expression vector for *ykfI*	[6]
pCA24N-*yafW*	Cm^{R}; expression vector for *yafW*	[6]
pCA24N-*yafW-ykfI*	Cm^{R}; expression vector for *yafW-ykfI*	This study
pCA24N-*ypjF*	Cm^{R}; expression vector for *ypjF*	[6]
pCA24N-*yfjZ*	Cm^{R}; expression vector for *yfjZ*	[6]
pCA24N-*yfjZ-ypjF*	Cm^{R}; expression vector for *yfjZ-ypjF*	This study

pCA24N-CP4-6-L5	Cm^R; expression vector for *yafX*, *ykfG*, *ykfH*, *yafW* and *ykfI* of prophage CP4-6	This study
pCA24N-CP4-57-R5	Cm^R; expression vector for *yfjX*, *yfjY*, *ypjJ*, *yfjZ* and *ypjF* of prophage CP4-57	This study
pBAD-*ykfI*	Amp^R; expression vector for *ykfI*	This study
pBAD-*ypjF*	Amp^R; expression vector for *ypjF*	This study
pBAD-*cbtA*	Amp^R; expression vector for *cbtA*	This study
pET28b-*yafW*	Km^R; expression vector for *yafW*	This study
pET28b-*yfjZ*	Km^R; expression vector for *yfjZ*	This study
pET28b-*cbeA*	Km^R; expression vector for *cbeA*	This study
pHGE	pHGE-P_{tac}, Km^R, IPTG inducible expression vector	[4]
pHGE-*ykfI*	Km^R; expression vector for *ykfI*	This study
pHGE-*ypjF*	Km^R; expression vector for *ypjF*	This study
pHGE-*cbtA*	Km^R; expression vector for *cbtA*	This study
pKT25-*zip*	Km^R; derived from pKT25. Sequence coding for the leucine zipper region of the GCN4 yeast protein. Positive control	[7]
pKT25-*ftsZ*	Km^R; expression vector for FtsZ	This study
pKT25-*mreB*	Km^R; expression vector for MreB	This study
pUT18C	Amp^R; derived from pUC19. Plac–MCS(*Hin*dIII–*Sph*I–*Pst*I–*Sal*I–*Xba*I–*Bam*HI–*Sma*I–*Kpn*I–*Sac*I–*Eco*RI)–T18	[7]
pUT18C-*zip*	Amp^R; derived from pUT18C. Sequence coding for the leucine zipper region of the GCN4 yeast protein. Positive control	[7]
pUT18C-*ykfI*	Amp^R; expression vector for *ykfI*	This study
pUT18C-*yafW*	Amp^R; expression vector for *yafW*	This study
pUT18C-*ypjF*	Amp^R; expression vector for *ypjF*	This study
pUT18C-*yfjZ*	Amp^R; expression vector for *yfjZ*	This study
pUT18C-*cbtA*	Amp^R; expression vector for *cbtA*	This study
pUT18C-*cbeA*	Amp^R; expression vector for *cbeA*	This study
pHERD20T-*ykfI*	Car^R; expression vector for *ykfI*	This study
pHERD20T-*ypjF*	Car^R; expression vector for *ypjF*	This study
pHERD20T-*cbtA*	Car^R; expression vector for *cbtA*	This study

Km^R, Cm^R, Amp^R and Car^R are resistance to kanamycin, chloramphenicol, ampicillin and carbenicillin, respectively.

Table S2. Minimum inhibitory concentrations (MICs) value for the BW25113 wild type strain and the deletion mutant strains.

Strains	Antibiotics（μg/ml）					
	Cip	Cm	Kan	Tet	PB	Fox
WT	0.05	6	6	1	0.5	4
Δ*ykfI*	0.05	4	6	1	0.5	4
Δ*yafW*	0.05	8	6	1	0.5	8
Δ*ypjF*	0.05	8	6	1	0.5	4
Δ*yfjZ*	0.05	8	6	1	0.5	8
Δ*cbtA*	0.1	4	8	1	0.5	4
Δ*cbeA*	0.1	4	8	1	0.5	4
Δ*ykfI*Δ*ypjF*Δ*cbtA*	0.1	4	8	1	0.5	4
Δ*yafW*Δ*yfjZ*Δ*cbeA*	0.1	4	8	1	0.5	4

Cip: Ciprofloxacin, Cm: Chloramphenicol, Kan: Kanamycin, Tet: Tetracycline, PB: Polymyxin B, Fox: Cefoxitin.

Table S3. Primers used in this study.

Primers	Sequences (5′-3′)[a]
	DNA sequencing
pCA24N-F	GATAACAATTTCACACAGAATT
pCA24N-R	GTCAGAGGTTTTCACCGTCATCA
pHGE-F	AGCTGTTGACAATTAATCATCG
pHGE-R	CACTTCTGAGTTCGGCATGG
pBAD-F	ATGCCATAGCATTTTTATCCA
pBAD-R	TCTGATTTAATCTGTATCAGG
T7-F	TAATACGACTCACTATAGGG
T7-R	TATGCTAGTTATTGCTCAG
pKT25-F	GCGAGGGCTATGTCTTCTACG
pKT25-R	GGGCTGGCTTAACTATGCGG
pUT18C-F	CGCATCTGTCCAACTTCCGC
pUT18C-R	CGCCAGGGTTTTCCCAGTCA
pHERD20T-F	ATCGCAACTCTCTACTGTTTCT
pHERD20T-R	TGCAAGGCGATTAAGTTGGGT
JC6-F	GGGACGTTCCGGATATCGC
JC6-R	CTTCTGGTTGCATGATTGTT
JC57-F	ATATCACAGGAGTCCTGGC
JC57-R	CCATTGTTGGTCCTAAAAGAAAAT
JC44-F	AGTTTTGGTCCGTATGTCC
JC44-R	TAACCGTTTTCAGCGGCCT
	Plasmids construction
pCA24N-yafW-ykfI-F	ACGC<u>GTCGAC</u>AGCAACCCTACCAGGGG
pCA24N-yafW-ykfI-R	CCC<u>AAGCTT</u>TCATCGTACTACGTTGTTACGGC
pCA24N-yfjZ-ypjF-F	ACGC<u>GTCGAC</u>AGCAACACCACATGGGG
pCA24N-yfjZ-ypjF-R	CCC<u>AAGCTT</u>TTATTTCACATTAGTTTTTAG
pCA24N-CP4-6-L5-F	ACGC<u>GTCGAC</u>ATGACAACACAGACGCAGCAC
pCA24N-CP4-6-L5-R	CCC<u>AAGCTT</u>TCATCGTACTACGTTGTTACGGC
pCA24N-CP4-57-R5-F	ACGC<u>GTCGAC</u>ATGACAACACAGACACAGTACG
pCA24N-CP4-57-R5-R	CCC<u>AAGCTT</u>TTATTTCACATTAGTTTTTAG
pHGE-ykfI-F	CCG<u>GAATTC</u>ATGAAAACTTTACCTGCAATAACTC
pHGE-ykfI-R	CCC<u>AAGCTT</u>TCATCGTACTACGTTGTTACGGC
pHGE-ypjF-F	CCG<u>GAATTC</u>ATGAACACTCTACCTGCTAC
pHGE-ypjF-R	CCC<u>AAGCTT</u>TTATTTCACATTAGTTTTTAGCAAGC
pHGE-cbtA-F	CCG<u>GAATTC</u>ATGAAAACATTACCTGTATTACCC
pHGE-cbtA-R	CCC<u>AAGCTT</u>TCATTTCGCCTCCGGATAC

pBAD-ykfI-F	CATGCCATGGGCAAAACTTTACCTGCAAT
pBAD-ykfI-R	CCCAAGCTTTCATCGTACTACGTTGTTACGGC
pBAD-ypjF-F	CATGCCATGGGCAACACTCTACCTGC
pBAD-ypjF-R	CCCAAGCTTTTATTTCACATTAGTTTTTAG
pBAD-cbtA-F	CATGCCATGGGCAAAACATTACCTGTATTACCC
pBAD-cbtA-R	CCCAAGCTTTCATTTCGCCTCCGGATAC
pET28b-yafW-F	TTTAAGAAGGAGATATACCATGGGCAGCAGCCATCATCATCATCATCACAGCAACC CTACCAGG
pET28b-yafW-R	GCTCGAGTGCGGCCGCAAGCTTTTAACGCTGAGTGGGG
pET28b-yfjZ-F	TTTAAGAAGGAGATATACCATGGGCAGCAGCCATCATCATCATCATCACAGCAACA CCACATG
pET28b-yfjZ-R	GCTCGAGTGCGGCCGCAAGCTTTTAGCGTTGAGTGGGG
pET28b-cbeA-F	TTTAAGAAGGAGATATACCGTGTCAGACACACTCCCCGGG
pET28b-cbeA-R	GCTCGAGTGCGGCCGCTTAATTTTTCATTTCGGGC
pUT18C-ykfI-F	CGGGGTACCGAAAACTTTACCTGCAAT
pUT18C-ykfI-R	CCGGAATTCTCATCGTACTACGTTGTTACGGC
pUT18C-yafW-F	CGGGGTACCGAGCAACCCTACCAGG GG
pUT18C-yafW-R	CCGGAATTCTTAACGCTGAGTGGGG
pUT18C-ypjF-F	CGGGGTACCGAACACTCTACCTGC
pUT18C-ypjF-R	CCGGAATTCTTATTTCACATTAGTTTTTAG
pUT18C-yfjZ-F	CGGGGTACCGAGCAACACCACATGGGG
pUT18C-yfjZ-R	CCGGAATTCTTAGCGTTGAGTGGGG
pUT18C-cbtA-F	CCGGAATTCTCATTTCGCCTCCGGATAC
pUT18C-cbtA-R	CGGGGTACCGAAAACATTACCTGTATTACCC
pUT18C-cbeA-F	CGGGGTACCGGTGTCAGACACACTCCCCGGG
pUT18C-cbeA-R	CCGGAATTCTTAATTTTTCATTTCGGGC
pKT25-FtsZ-F	CTAGAGGATCCCCGGGTACCTTTTGAACCAATGGAACTTACC
pKT25-FtsZ-R	GAATTCTTAGTTACTTAGTTAATCAGCTTGCTTACGCAG
pKT25-MreB-F	CTAGAGGATCCCCGGGTACCTTTGAAAAAAATTTCGTGGCATGTT
pKT25-MreB-R	GAATTCTTAGTTACTTAGTTACTCTTCGCTGAACAGGTC
pHERD20T-YkfI-F	CCGGAATTCGATGAAAACTTTACCTGCAATAACTC
pHERD20T-YkfI-R	CCCAAGCTTTCATCGTACTACGTTGTTACGGC
pHERD20T-YpjF-F	CCGGAATTCGATGAACACTCTACCTGCTAC
pHERD20T-YpjF-R	CCCAAGCTTTTATTTCACATTAGTTTTTAGCAAGC
pHERD20T-CbtA-F	CCGGAATTCGATGAAAACATTACCTGTATTACCC
pHERD20T-CbtA-R	CCCAAGCTTTCATTTCGCCTCCGGATAC
	qRT-PCR
rrsG-F	TATTGCACAATGGGCGCAAG
rrsG-R	ACTTAACAAACCGCTGCGT
q-ykfI-F	CATTCTGCAATGAGGCTGTGA
q-ykfI-R	CAGCCCGGAGATAAGGAGATT
q-ypjF-F	CAGTGGCTGTCTGGCAAATG
q-ypjF-R	TCCACCAGAAAATTGACTGCAT
q-cbtA-F	TTCTCGCCCGTCTCCTGTT
q-cbtA-R	ACCGCATCACACAGTGAAATG
q-yafW-F	CCTGCAGCGGGAGATTACAC
q-yafW-R	GAGGATAAAGTGCGGGAATGTT
q-yfjZ-F	GGCCTGCAGCGAGATATCAC
q-yfjZ-R	TGGCTGATAAAATGTGGAAATACC
q-cbeA-F	GCTGCTGATGAAACAACTGGAA
q-cbeA-R	GTCGGATAAACAGCCAGATAAACG

[a], restriction sites included in oligonucleotide sequences are underlined.

Supplementary Video 1. Over-production of toxin YkfI cause "lemon-shaped" cells to further form "gourd-shaped" cells. This video was adjusted to 6× in speed to reduce time duration. Cell burst was observed at 1 s, 4 s, 11 s and 17 s.

References

1. Baba, T.; Ara, T.; Hasegawa, M.; Takai, Y.; Okumura, Y.; Baba, M.; Datsenko, K.A.; Tomita, M.; Wanner, B.L.; Mori, H. Construction of *Escherichia coli* K-12 in-frame, single-gene knockout mutants: The Keio collection. *Mol. Syst. Bio.l* **2006**, *2*, doi:10.1038/msb4100050.
2. Obrien, A.D.; Lively, T.A.; Chen, M.E.; Rothman, S.W.; Formal, S.B. *Escherichia coli* O157:H7 strains associated with hemorrhagic colitis in the United States produce a Shigella dysenteriae 1 (SHIGA) like cytotoxin. *Lancet* **1983**, *1*, 702–702.
3. Stover, C.K.; Pham, X.Q.; Erwin, A.L.; Mizoguchi, S.D.; Warrener, P.; Hickey, M.J.; Brinkman, F.S.L.; Hufnagle, W.O.; Kowalik, D.J.; Lagrou, M., et al. Complete genome sequence of *Pseudomonas aeruginosa* PAO1, an opportunistic pathogen. *Nature* **2000**, *406*, 959–964.
4. Shi, M.; Wu, L.; Xia, Y.; Chen, H.; Luo, Q.; Sun, L.; Gao, H. Exoprotein Production Correlates with Morphotype Changes of Nonmotile *Shewanella oneidensis* Mutants. *J. Bacteriol.* **2013**, *195*, 1463–1474.
5. Datsenko, K.A.; Wanner, B.L. One-step inactivation of chromosomal genes in *Escherichia coli* K-12 using pcr products. *Proc. Natl. Acad. Sci. USA* **2000**, *97*, 6640–6645.
6. Kitagawa, M.; Ara, T.; Arifuzzaman, M.; Ioka-Nakamichi, T.; Inamoto, E.; Toyonaga, H.; Mori, H. Complete set of ORF clones of *Escherichia coli* ASKA library (A complete set of *E. coli* K-12 ORF archive): Unique resources for biological research. *DNA Res.* **2005**, *12*, 291–299.
7. Karimova, G.; Pidoux, J.; Ullmann, A.; Ladant, D. A bacterial two-hybrid system based on a reconstituted signal transduction pathway. *Proc. Natl. Acad. Sci .USA* **1998**, *95*, 5752–5756.
